# Psychiatric Disorders in Adolescents Attending Psychiatry Outpatient Department in a Tertiary Care Hospital: A Descriptive Cross-sectional Study

**DOI:** 10.31729/jnma.5580

**Published:** 2021-06-30

**Authors:** Neelam Joshi, Asim Shrestha, Deepika Karki, Pradip Man Singh

**Affiliations:** 1 Department of Psychiatry, Nepal Medical College and Teaching Hospital, Jorpati, Kathmandu, Nepal; 2 Nepal Medical College and Teaching Hospital, Jorpati, Kathmandu, Nepal

**Keywords:** *adolescent*, *ICD-10*, *morbidity*, *mortality*, *psychiatric disorders*

## Abstract

**Introduction:**

Adolescence is the time when most mental illness begins most of the time these problems are overlooked leading to increased morbidity and mortality. The study is undertaken to find out the prevalence of psychiatric disorders in adolescents attending psychiatry outpatient department in a tertiary hospital.

**Methods:**

A descriptive cross-sectional study was done from 1^st^ December 2019 to 29^th^ November 2020 at a tertiary care centre of Kathmandu. Ethical approval (Ref No.: 012-076/077) was taken from the Institution Review Committee. A semi-structured proforma was used for the socio-demographic profile of patients and International Classification of Diseases-10 was used to make the diagnosis. Data were analyzed using Statistical Package for the Social Sciences version 16.

**Results:**

A total of 174 adolescents were included in the study. Out of the total cases 141 (81.03%) (75.1-86.8 at 95% Confidence Interval) were diagnosed with psychiatric disorders, among them 73 (51.77%) were males and 68 (48.23%) were females. The common diagnoses were anxiety disorders 63 (36.20%), mood disorders 34 (19.54%), psychotic disorders 26 (14.94%), substance use disorders 8 (4.59%), non-organic sleep disorders 5 (2.87%), behavioral and emotional disorders 3 (1.72%) and mental retardation 2 (1.149%).

**Conclusions:**

Prevalence of psychiatric illnesses is high in the adolescent population compared to the similar studies.

## INTRODUCTION

Adolescence is the transitional period marked by physiological signs and surging hormones.^1^ World Health Organization (WHO) defines adolescence as the age between 10-19 years.^2^ One in six people in the world is adoleascents.^3^ 16% of the global burden of disease in adolescents is due to mental problems as adolescence is marked immense turmoil.^2,3^

One in five adolescents experiences a mental health disorder each year.^4^ Half of all the mental health conditions start by the age of 14 years but most are undetected.^3^ 24.19% of the total population in Nepal constitute 10-19 years age group.^5^ Physical, emotional and social changes predispose adolescents to mental health problems.^6^ Global burden of disease study in 10-24 years old, revealed the leading cause of the years of life lost due to disability is neuropsychiatric disorders (45%).^7^ Hence, adolescence is the time of increased risk of psychiatric illness.^8^

The aim of the study is to find out the prevalence of psychiatric disorders in adolescents attending psychiatry outpatient department in a tertiary care hospital.

## METHODS

This is a descriptive cross-sectional study carried out in Nepal Medical College and Teaching Hospital, Jorpati, Kathmandu for a duration of one year from December 1^st^, 2019 to November 29^th^, 2020. Ethical clearance was obtained from Institution Review Committee (IRC-NMCTH) before starting the study with an archived approval number 012-076/077. After explaining the objective and plan of study and fulfilling inclusion and exclusion criteria, informed written consent was taken from the patient and/or the guardian. Patients in the age group of 10-19 years were included and patients with serious medical illness were excluded from the study. The cases either presented to the Psychiatric OPD or were referred from other OPDs for consultation.

A self-designed proforma containing questions on socio-demographic data of adolescents was prepared separately and pretested in 17 cases before final administration to enrolled cases. Following this, a detailed clinical interview, mental status examination of the adolescents was done. The diagnosis of psychiatric disorders was made as per the ICD 10 criteria.

Convenience sampling was done and the minimum sample size required for the study was calculated as follow:

$$\begin{array}{ll} \text{n} & \text{= }{\text{Z}}^{\text{2}}\times (\text{p}\times \text{q)}/{\text{e}}^{\text{2}}\\ & \text{= }{\left(1.96\right)}^{\text{2}}\times 0.33\times 0.67/{(\text{0.05)}}^{\text{2}}\\ & \text{= }174 \end{array}$$

Where,

n = minimum sample size
z = 1.96 for C.I = 95%
p = prevalence, 33.33 % among 10-19 age group.^9^
q = (1-p)
e = margin of error, 5%
Total sample size was calculated to be 174.

Selection bias has been minimized as possible and the collected data was analyzed using SPSS 16 and descriptive analysis was done. Point estimate at 95% Confidence Interval was done for binary data along with analysis for frequency and proportion.

## RESULTS

Out of 174 adolescents (10-19 years) attending the psychiatric outpatient department (OPD), 141 (81.03%) (75.1-86.8 at 95% CI) were diagnosed with a psychiatric disorder. The mean age of patients was 16.77 years±1.99. Among the study, 93 (53.45%) were females and 81 (46.55%) were males. The prevalence of psychiatric disorders was the highest in the age group 14-16years. Gender-wise distribution showed a higher prevalence in the males 73 (51.77%) as compared to females 68 (48.23%).

The commonest psychiatric diagnosis was anxiety disorders 63 (36.20%), followed by mood disorders 34 (19.54%), psychotic disorders 26 (14.94%), substance abuse disorders were found in 8 (4.59%) adolescents, non-organic sleep disorders in 5 (2.87%), behavioral and emotional disorders in 3 (1.72%) and mental retardation in 2 (1.149%) (Table 1).

**Table 1:** Distribution of respondents on the basis of ICD-10 DCR diagnosis.

ICD-10 Diagnostic Criteria	n (%)
Mental and behavioral disorders due to psychoactive substance use	
Alcohol dependence syndrome	1 (0.57)
Cannabis dependence syndrome	2 (1.15)
Cannabis induced psychotic disorder	1 (0.57)
Polysubstance dependence syndrome	4 (2.3)
Total	8 (4.59)
Schizophrenia, schizotypal and delusional disorders	
Schizophrenia	12 (6.9)
Acute and transient psychotic disorders	5 (2.87)
Unspecified nonorganic psychotic disorders	9 (5.17)
Total	26 (14.94)
Mood disorders	
Mania without psychotic symptoms	2 (1.1)
Bipolar affective disorder	8 (4.6)
Mild depressive episode	6 (3.5)
Moderate depressive episode	14 (8.05)
Severe depressive episode without psychotic symptoms	1 (0.57)
Severe depressive episode with psychotic symptoms	2 (1.1)
Recurrent depressive disorder	1 (0.57)
Total	34 (19.54)
Neurotic, stress related and somatoform disorder	
Panic disorder	2 (1.15)
GAD	2 (1.15)
Anxiety disorder, unspecified	13 (7.5)
OCD	5 (2.87)
Adjustment disorders	18 (10.34)
Dissociative disorders	23 (13.21)
Total	63 (36.20)
Nonorganic sleep disorders	
Non-organic insomnia	5 (2.87)
Total	5 (2.87)
Mental retardation	
Moderate mental retardation	2 (1.15)
Total	2 (1.15)
Behavioral and emotional disorders with onset usually occurring in childhood and adolescence	
Hyperkinetic disorder	1 (0.57)
Conduct disorder	1 (0.57)
Nonorganic enuresis	1 (0.57)
Total	3 (1.72)

The majority of patients diagnosed with a psychiatric disorder were in the age group years 17-19 yrs 109 (60.28%) with a male preponderance 56 (51.77%). Male to female ratio 1.07:1 and the majority of them were single 165 (95.04%). Of the total patients, 97 (56.03%) belonged to low Socio-economic status, and 88 (51.06%) from inside Kathmandu valley. 62 (36.17%) of patients had completed intermediate education and 17 (9.93%) were working. Amongst those diagnosed, 114 (65.96%) were Hindu by religion, 92 (53.19%) belonged to a nuclear family, 161 (92.91%) lived with both parents, 51 (29.79%) had a history of migration and 38 (21.99%) had a history of abuse. Twenty eight (16.31%) had a history of substance use and 19 (11.35%) had a family history of psychiatric illness. 22 (12.77%) experienced suicidal ideations while only 12 (7.09%) attempted suicide. 107 (61.70%) experienced stressors before illness onset. In our study, the distribution of different socio-demographic variables and different psychiatric disorders were found to be as follows (Table 2,3).

**Table 2:** Distribution of respondents on the basis of sociodemographic variables.

Characteristics	Category	n (%)
1. Sex	Male	81 (46.55)
	Female	93 (53.45)
2. Age	10-13	13 (7.47)
	14-16	52 (29.89)
	17-19	109 (62.64)
3. Education	Illiterate	2 (1.15)
	Under SLC	68 (39.08)
	SLC	37 (21.26)
	Intermediate and above	67 (38.51)
4. Marital status	Single	167 (95.98)
	Married	4 (2.30)
	In a relationship	3 (1.72)
5. Occupation	Unemployed	30 (17.24)
	Student	120 (68.97)
	Labor	4 (2.30)
	Semiskilled	11 (6.32)
	Skilled	9 (5.17)
6. Religion	Hindu	117 (67.24)
	Buddhist	55 (31.61)
	Others	2 (1.15)
7. Address	Inside KTM valley	94 (54.02)
	Outside KTM valley	80(45.9)
8. SES	Low	87 (50.00)
	Middle	71 (40.80)
	Upper	16 (9.20)
9. Family	Nuclear	90 (51.72)
	Joint	84 (48.28)
10. Family status	Both parents	159 (91.38)
	Single parent	15 (8.62)
11. H/o migration	Yes	51 (29.31)
	No	123 (70.69)
12. H/o abuse	Yes	37 (21.26)
	No	137 (78.74)
13. H/o Substance abuse	Yes	22 (12.64)
	No	152 (87.36)
14. F/H/O psychiatric	Yes	16 (9.20)
illness	No	158 (90.80)
15. Stressor	Present	76 (43.68)
	Absent	98 (56.32)
Mean age: 16.77 years ± 1.99		

**Table 3:** Psychiatric disorders according to ICD-10 DCR and different variables.

Socio-demographic variables		ICD-10 DCR							
		F 10	F 20	F 30	F 40	F 55	F 71	F 90	n (%)
Sex	Male	8 (100)	19 (73.07)	19 (55.88)	19 (30.15)	4 (80)	2 (100)	2 (56.66)	73 (51.77)
	Female	0 (0)	7 (26.92)	15 (44.11)	44 (69.84)	1 (20)	0 (0)	1 (33.33)	68 (48.23)
	Total	8 (100)	26 (100)	34 (100)	63 (100)	5 (100)	2 (100)	3 (100)	141 (100)
Age	10-13	0 (0)	0 (0)	3 (8.82)	3 (4.76)	1 (20)	0	1 (33.33)	8 (5.67)
	14-16	2 (25)	8 (30.76)	7 (20.58)	28 (44.44)	1 (20)	1 (50)	1 (33.33)	48 (34.04)
	17-19	6 (75)	18 (69.23)	24(70.58)	32 (50.79)	3 (60)	1 (50)	1(33.33)	85 (60.28)
	Total	8 (100)	26 (100)	34 (100)	63 (100)	5 (100)	2 (100)	3 (100)	141 (100)
Education	Illiterate	0 (0)	0 (0)	0 (0)	0 (0)	0 (0)	2 (100)	0 (0)	2 (1.42)
	<SLC	6 (75)	7 (26.92)	13 (38.23)	25 (39.68)	2 (40)	0	2 (66.66)	55 (39.01)
	SLC	1 (12.5)	9 (34.61)	9 (26.47)	12 (19.04)	2 (40)	0 (0)	0 (0)	33 (23.40)
	XII and above	1 (12.5)	10 (38.46)	12 (35.29)	26 (41.26)	1 (20)	0 (0)	1 (33.33)	51 (36.17)
	Total	8 (100)	26 (100)	34 (100)	63 (100)	5 (100)	2 (100)	3 (100)	141 (100)
Marital status	Single	8 (100)	26 (100)	30 (88.23)	60 (95.23)	5 (100)	2 (100)	3 (100)	134 (95.04)
	In a relation			2(5.88)	1(1.58)				3 (2.13)
	Married			2 (5.88)	2 (3.17)				4 (2.84)
	Total	8 (100)	26 (100)	34 (100)	63 (100)	5 (100)	2 (100)	3 (100)	141 (100)
Occupation	Unemployed	8 (100)	23 (88.46)	28 (82.35)	55 (87.30)	4 (80)	2 (100)	3 (100)	123 (87.23)
	Labor			2(5.88)	2 (3.17)				4 (2.84)
	Semiskilled		3 (11.53)	4 (11.76)	1 (1.58)	1(20)			9 (6.38)
	Skilled				5 (7.93)				5 (3.55)
	Total	8 (100)	26 (100)	34 (100)	63 (100)	5 (100)	2 (100)	3 (100)	141 (100)
Religion	Hindu	5 (62.5)	18 (69.23)	23 (67.64)	42 (66.66)	3 (60)		2 (66.66)	93 (65.96)
	Buddhist	3 (37.5)	8 (30.77)	11 (32.35)	19 (30.15)	2 (40)	2 (100)	1 (33.33)	46 (32.62)
	Others				2 (3.17)				2 (1.42)
	Total	8 (100)	26 (100)	34 (100)	63 (100)	5 (100)	2 (100)	3 (100)	141 (100)
Address	Inside KTM valley	4 (50)	18 (69.23)	13 (38.23)	30 (47.61)	4 (80)	1 (50)	2 (66.66)	72 (51.06)
	Outside KTM valley	4 (50)	8 (30.76)	21 (61.76)	33 (52.38)	1 (20)	1 (50)	1 (33.33)	69 (48.94)
	Total	8 (100)	26 (100)	34 (100)	63 (100)	5 (100)	2 (100)	3 (100)	141 (100)
Socio economic status	Low	5 (62.5)	17 (65.38)	21 (61.76)	31 (49.20)	3 (60)	1 (50)	1 (33.33)	79 (56.03)
	Middle	3 (37.5)	9 (34.61)	13 (38.23)	31 (49.20)	2 (40)	1 (50)	2 (66.66)	61 (43.26)
	Upper				1 (1.58)				1 (0.71)
	Total	8 (100)	26 (100)	34 (100)	63 (100)	5 (100)	2 (100)	3 (100)	141 (100)
Family	Nuclear	7 (87.5)	11 (42.30)	17 (50)	33 (52.38)	3 (60)	2 (100)	2 (66.66)	75 (53.19)
	Joint	1 (12.5)	15 (57.69)	17 (50)	30 (47.61)	2 (40)		1 (33.33)	66 (46.81)
	Total	8 (100)	26 (100)	34 (100)	63 (100)	5 (100)	2 (100)	3 (100)	141 (100)
Family status	Both parents	7 (87.5)	26 (100)	28 (82.35)	62 (98.41)	5 (100)	2 (100)	1 (33.33)	131 (92.91)
	One parent	1(12.5)		6 (17.64)	1 (1.58)			2 (66.66)	10 (7.09)
	Total	8 (100)	26 (100)	34 (100)	63 (100)	5 (100)	2 (100)	3 (100)	141 (100)
h/o migration	Yes	2 (25)	2 (7.69)	13 (38.23)	23 (36.50)		1 (50)	1 (33.33)	42 (29.79)
	No	6 (75)	24 (92.30)	21 (61.76)	40 (63.49)	5 (100)	1 (50)	2 (66.66)	99 (70.21)
	Total	8 (100)	26 (100)	34 (100)	63 (100)	5 (100)	2 (100)	3 (100)	141 (100)
h/o abuse	Yes	1 (12.5)	2 (7.69)	8 (23.53)	20 (31.74)				31 (21.99)
	No	7 (87.5)	24 (92.30)	26 (76.47)	43 (68.25)	5 (100)	2 (100)	3 (100)	110 (78.01)
	Total	8 (100)	26 (100)	34 (100)	63 (100)	5 (100)	2 (100)	3 (100)	141 (100)
History of substance use	Yes	8 (100)	6 (23.07)	2 (5.88)	5 (7.93)	2 (40)			23 (16.31)
	No		20 (76.92)	32 (94.11)	58 (92.06)	3 (60)	2 (100)	3 (100)	118 (83.69)
	Total	8(100)	26(100)	34(100)	63(100)	5(100)	2(100)	3(100)	141 (100)
Family history of psychiatric illness	Yes	2 (25)	1 (3.84)	2 (5.88)	11 (17.46)				16 (11.35)
	No	6 (75)	25 (96.15)	32 (94.11)	52 (82.53)	5 (100)	2 (100)	3 (100)	125 (88.65)
	Total	8 (100)	26 (100)	34 (100)	63 (100)	5 (100)	2 (100)	3 (100)	141 (100)
SI/SP	Yes			9 (26.47)	9 (14.28)				18 (12.77)
	No	8 (100)	26 (100)	25 (73.52)	54 (85.71)	5 (100)	2 (100)	3 (100)	123 (87.23)
	Total	8 (100)	26 (100)	34 (100)	63 (100)	5 (100)	2 (100)	3 (100)	141 (100)
Suicidal Attempt	Yes	1 (12.5)		4 (11.76)	5 (7.93)				10 (7.09)
	No	7 (87.5)	26 (100)	30 (88.23)	58 (92.06)	5 (100)	2 (100)	3 (100)	131 (92.91)
	Total	8 (100)	26 (100)	34 (100)	63 (100)	5 (100)	2 (100)	3 (100)	141 (100)
Stressor	Present	2 (25)	9 (34.61)	22 (64.70)	50 (79.36)	4 (80)			87 (61.70)
	Absent	6 (75)	17 (65.38)	12 (35.29)	13 (20.63)	1 (20)	2 (100)	3 (100)	54 (38.30)
	Total	8 (100)	26 (100)	34 (100)	63 (100)	5 (100)	2 (100)	3 (100)	141 (100)

**Figure 1:**
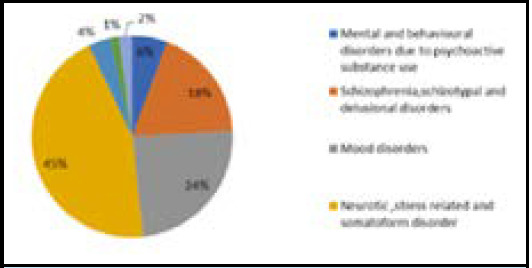
Distribution of psychiatric disorder in adolescent patients.

The overall distribution from our study showed that the commonest psychiatric disorder was neurotic, stress related and somatoform disorders followed by mood disorders and Schizophrenia, schizotypal and delusional disorders respectively. Other disorders such as mental and behavioral disorders due to psychoactive use and mental retardation were found to be comparatively less common (Figure 1).

## DISCUSSION

In our study, the mean age of the patients under study was 16.77±1.99 years. Eighty-one cases were male and 93 cases female patients. Here females seem to attend OPD more since adolescent females experience more mental issues than males which is also supported by other studies.^8^

In our study, 81.03% of the adolescents attending the Psychiatric department were diagnosed with a psychiatric disorder. The result was significantly higher than other studies. It could be because the adolescents visiting the OPD were already experiencing some problems. It could also be pointing towards the growing trend of mental problems in this age group. However, this is not representative of the community.

Worldwide, many studies have been conducted on the prevalence of psychiatric comorbidities among adolescents which is found to be variable. A review of studies done in 20 countries, conducted over 40 years, reported a very wide range in prevalence of mental disorders in adolescents.^10-14^ Similarly, studies conducted on the adolescents in various parts of India reported a prevalence ranging from 1.81% to 22.17%.^10,15-17^ As per a report on a pilot study of the National Mental Health Survey in Nepal, the current prevalence of any form of mental disorders among adolescents is 12.9% with 11.2% in the age group of 13-17years.^18^ Other studies in Nepal state that 12.9-30% of adolescents had psychosocial problems.^5,19,20^

In our findings anxiety disorders were found to be the commonest, this is similar to the meta-analysis done by Polanczyk, et al. 6.5%,^21^ on the worldwide prevalence of mental disorders in children and adolescents, similar findings were recorded by Pillai, et al. 1%,^10^ Roberts 6.9%,^22^ Mishra 15%,^23^ Kessler, et al.^24^ In a study done in Dang district, (46.5%) of the secondary school adolescents had anxiety.^25^

Our study showed mood disorder to be the second most common disorder. Balgir, et al.^17^ reported mood disorders to be the most common and Lynch, et al. 4.5%,^13^ reported affective disorders to be the most prevalent in their studies. Kessler, et al.^24^ and Merikangas, et al. 14.3%,^12^ reported mood disorder to be the third most common. Variability in the results may be due to differences in sample size, different study methods used.

There have been various studies demonstrating the increasing trend of suicidal tendencies among adolescents. Lynch, et al.^13^ reported 1.9% of adolescents with suicidal ideations, McMahon E, et al.^26^ from a study in Ireland reported 7% suicidal ideation and 3.6% of attempted suicide. In Nepal, the National Mental Health survey reported suicidality in 8.7% of adolescents.^18^ In our study, suicidal ideations were seen in 12.77% of adolescents while the suicidal attempt was seen in 7.09%.

Kelleher, et al.^27^ noted 17% of adolescents aged (9-12years) and 7.5% aged (13-18years) were diagnosed with psychosis. Large population-based studies surveying psychotic symptoms among adolescents found rates of 9-14% in interview-based studies.^28^ Similarly, in our study, schizophrenia spectrum disorders were found to be 14.94%.

Our study found Substance abuse disorders (SUD) to be 4.59% which is similar to Roberts, et al.^22^ 5.3%, other studies reported SUD as Merikangas, et al.^12^ 11.4%, Kessler, et al. ^22^ as the fourth common disorders among adolescents. Reinherz, et al.^29^ reported drug abuse in 9.8% while a pilot study in Nepal,^18^ substance use, in adolescents, was found in 0.7%. Studies have reported variable results, which could be due to the different geographical populations under study and differences in the nature of the study.

The prevalence of sleep disorders was reported to be 1-43% in international studies.^30^ Ipsiroglu, et al.^30^ reported 12% school children (11-15) years with sleep problems while 13.1% of adolescents were diagnosed with non-organic sleep disorders by Kaur, et al.^31^ In our study, non-organic sleep disorder was found in 2.87% of adolescents, which is within the range mentioned in international studies.

Kessler, et al.^24^ reported behavior disorder as the second most common in their study. Merikangas, et al.^12^ reported 19.1% of adolescents with behavior disorders. Wagner, et al.^14^ stated 5.2% of adolescents with behavior disorder while a study by Roberts, et al.^22^ reported 6.5%. Pillai, et al.^10^ stated 0.4% of adolescents are diagnosed with a behavior disorder. All studies have shown different results. Similarly, in our study, behavioral disorders were found to be 1.72% in adolescents which might be due to our sample size being smaller.

Wagner, et al.^14^ showed 9.3% of adolescents diagnosed with neurodevelopment disorders. 6.8 % of adolescents were diagnosed with neurodevelopment disorders in a study by Ogbonna, et al.^32^ In our study, 1.149% of adolescents were diagnosed with Intellectual disability. This low percentage could be because most neurodevelopment disorders are diagnosed in childhood itself, hence fewer remain to be diagnosed up until adolescence.

The limitation of this study is its small number of cases, convenience sampling, and study done at a single center. We need larger studies with larger sample size, scientific sampling, and multicentered studies to understand the magnitude of the problem in the population in the future.

## CONCLUSIONS

Worldwide, a large number of studies have been reported in the medical literature pointing towards the increased prevalence of psychiatric disorders in adolescent patients. However, in Nepal, there is a scarcity of studies that explore the arena of adolescent mental health. Our study concluded that the burden of psychiatric disorders is increasing in the adolescent population and hence needs to be addressed. The findings of our study intend to suggest that the prevalence of psychiatric illnesses is high in the adolescent population compared to similar studies. This emphasizes the need for screening all adolescents and to educate the family and guardians regarding the mental illnesses and warning signs, for early detection and treatment.

## References

[1] Sadock BJ, Sadock VA, Ruiz P (2017). Kaplan and Sadock's Comprehensive Textbook of Psychiatry.

[2] Pathak R, Sharma RC, Parvan UC, Gupta BP, Ojha RK, Goel N (2011). Behavioural and emotional problems in school going adolescents. Australas Med J.

[3] World Health Organisation. (2020). Adolescent mental health [Internet].

[4] United Nations International Children's Emergency Fund. (2019). Mental health [Internet].

[5] Timalsina M, Kafle M, Timalsina R (2018). Psychosocial Problems among School Going Adolescents in Nepal. Psychiatry J.

[6] Carpentier C, Carvacho P, Me A, Crean C, Davis P, Bisogno E (2018). World Drug Report 2018.

[7] Tasman A, Kay J, Liberman JA, First MB, Riba M (2015). Psychiatry.

[8] Lopes CS, Abreu Gde A, dos Santos DF, Menezes PR, de Carvalho KM, Cunha Cde F, de Vasconcellos MT, Bloch KV, Szklo M (2016). ERICA: prevalence of common mental disorders in Brazilian adolescents. Rev Saude Publica.

[9] Hossain MM, Purohit N (2019). Improving child and adolescent mental health in India: Status, services, policies, and way forward. Indian J Psychiatry.

[10] Pillai A, Patel V, Cardozo P, Goodman R, Weiss HA, Andrew G (2008). Non-traditional lifestyles and prevalence of mental disorders in adolescents in Goa, India. Br J Psychiatry.

[11] Wittchen HU, Nelson CB, Lachner G (1998). Prevalence of mental disorders and psychosocial impairments in adolescents and young adults. Psychol Med.

[12] Merikangas KR, He JP, Burstein M, Swanson SA, Avenevoli S, Cui L, Benjet C, Georgiades K, Swendsen J (2010). Lifetime prevalence of mental disorders in U.S. adolescents: results from the National Comorbidity Survey Replication-Adolescent Supplement (NCS-A). J Am Acad Child Adolesc Psychiatry.

[13] Lynch F, Mills C, Daly I, Fitzpatrick C (2006). Challenging times: prevalence of psychiatric disorders and suicidal behaviours in Irish adolescents. J Adolesc.

[14] Wagner G, Zeiler M, Waldherr K, Philipp J, Truttmann S, Dur W, Treasure JL, Karwautz AFK (2017). Mental health problems in Austrian adolescents: a nationwide, two-stage epidemiological study applying DSM-5 criteria. Eur Child Adolesc Psychiatry.

[15] Pahwa MG, Sidhu BS, Balgir RS (2019). A study of psychiatric morbidity among school going adolescents. Indian J Psychiatry.

[16] Faizi N, Azmi SA, Ahmad A, Shah MS (2016). Assessment of psychological problems in schoolgoing adolescents of Aligarh. Ind Psychiatry J.

[17] Balgir RS, Sidhu BS, Garg M, Wats A, Sohal S (2016). Distribution of psychiatric morbidity among school going adolescents in a district of North India. Int'l J Med Res Health Sc.

[18] A Report on pilot study of National Mental Health Survey. (2018).

[19] Sharma B, Rai MK, Sharma A, Karki S (2019). Emotional and Behavioral Problems among Adolescents in Pokhara City in Nepal. J Nepal Health Res Counc.

[20] Bista B, Thapa P, Sapkota D, Singh SB, Pokharel PK (2016). Psychosocial Problems among Adolescent Students: An Exploratory Study in the Central Region of Nepal. Front Public Health.

[21] Polanczyk GV, Salum GA, Sugaya LS, Caye A, Rohde LA (2015). Annual research review: A meta-analysis of the worldwide prevalence of mental disorders in children and adolescents. J Child Psychol Psychiatry.

[22] Roberts RE, Roberts CR, Xing Y (2007). Rates of DSM-IV psychiatric disorders among adolescents in a large metropolitan area. J Psychiatr Res.

[23] Mishra SK, Srivastava M, Tiwary NK, Kumar A (2018). Prevalence of depression and anxiety among children in rural and suburban areas of Eastern Uttar Pradesh: A cross-sectional study. J Family Med Prim Care.

[24] Kessler RC, Avenevoli S, Costello EJ, Georgiades K, Green JG, Gruber MJ, He JP, Koretz D, McLaughlin KA, Petukhova M, Sampson NA, Zaslavsky AM, Merikangas KR (2012). Prevalence, persistence, and sociodemographic correlates of DSM-IV disorders in the National Comorbidity Survey Replication Adolescent Supplement. Arch Gen Psychiatry.

[25] Adhikari C, Bhandari KP (2015). Prevalence and factors associated with anxiety disorders among secondary school adolescents of Dang District, Nepal. J Gandaki Med Coll.

[26] McMohan E, O'Regan G, Corcoran P, Arensman E, Cannon M, Williamson E (2017). Young lives in Ireland. A school based study of mental health and suicide prevention.

[27] Kelleher I, Connor D, Clarke MC, Devlin N, Harley M, Cannon M (2012). Prevalence of psychotic symptoms in childhood and adolescence: a systematic review and meta-analysis of population-based studies. Psychol Med.

[28] Stevens JR, Prince JB, Prager LM, Stern TA (2014). Psychotic disorders in children and adolescents: a primer on contemporary evaluation and management. Prim Care Companion CNS Disord.

[29] Reinherz HZ, Giaconia RM, Lefkowitz ES, Pakiz B, Frost AK (1993). Prevalence of psychiatric disorders in a community population of older adolescents. J Am Acad Child Adolesc Psychiatry.

[30] Ipsiroglu OS, Fatemi A, Werner I, Tiefenthaler M, Urschitz MS, Schwarz B (2001). Häufigkeit von Schlafstörungen bei Schulkindern zwischen 11 und 15 Jahren [Prevalence of sleep disorders in school children between 11 and 15 years of age]. Wien Klin Wochenschr.

[31] Kaur S, Thapar SK, Shandilya Vui (2015). The prevalence of psychiatric morbidity among school children. Int'l J Med Dental Sc.

[32] Ogbonna PN, Iheanacho PN, Ogbonnaya NP, Mbadugha CJ, Ndubuisi I, Chikeme PC (2020). Prevalence of mental illness among adolescents (15-18 years) treated at Federal Neurospsychiatric Hospital, Enugu Nigeria, from 2004 to 2013. Arch Psychiatr Nurs.

